# Clozapine‐Induced Gastrointestinal Hypoperfusion Leading to Bowel Ischemia and Secondary Atrial Fibrillation: A Case Report

**DOI:** 10.1155/crps/8484558

**Published:** 2026-09-10

**Authors:** Shreya Kumar, Meera Chatterjee

**Affiliations:** ^1^ Temple University Lewis Katz School of Medicine, Philadelphia, Pennsylvania, USA, temple.edu

**Keywords:** atrial fibrillation, bowel ischemia, clozapine, hypoperfusion, schizophrenia

## Abstract

**Introduction:**

Clozapine is a highly effective antipsychotic for treatment‐resistant schizophrenia but carries a risk of rare and serious adverse events. This case highlights a potentially fatal episode of clozapine‐induced gastrointestinal hypoperfusion leading to bowel ischemia and secondary atrial fibrillation in a patient with chronic schizophrenia.

**Main Symptoms and Clinical Findings:**

A middle‐aged male with longstanding schizophrenia presented with abdominal pain, vomiting, nausea, and persistent tachycardia. He was diagnosed with bowel ischemia from hypoperfusion and new‐onset atrial fibrillation.

**Intervention and Outcome:**

Clozapine was discontinued. The patient underwent emergency laparotomy and supportive ICU care. His psychiatric management was shifted to haloperidol and olanzapine, and he returned close to his psychiatric baseline over time.

**Conclusion:**

This case emphasizes the importance of monitoring for rare gastrointestinal complications associated with clozapine, including hypoperfusion‐mediated bowel ischemia, and supports early recognition and prompt discontinuation to reduce morbidity and mortality.

## 1. Introduction

Clozapine remains the gold standard for treatment‐resistant schizophrenia due to its efficacy in reducing suicidality and behavioral disturbances [1]. However, it is not considered a first‐line antipsychotic because of its significant cardiovascular, metabolic, and hematologic adverse effect profile [2–4]. Its use is therefore generally reserved for refractory cases and is associated with a broad adverse effect profile, including rare gastrointestinal complications such as paralytic ileus and bowel ischemia [5]. Risk factors for such gastrointestinal complications include older age, male gender, patients in the first 4 months of treatment, coprescription of constipating agents, higher daily dose of clozapine, and previous gastrointestinal complication [6]. This case contributes to the limited but growing literature on clozapine‐induced bowel ischemia and highlights a rare complication involving secondary atrial fibrillation. Notably, this case specifically reports a hypoperfusion‐mediated etiology, distinct from the more commonly reported hypomotility‐driven presentations in the existing literature [5, 6].

## 2. Patient Information

• Demographics and history: Middle‐aged male with a history of schizophrenia, hypertension, and substance use disorder.
• Psychiatric medication history: Previously trialed multiple antipsychotics including aripiprazole, ziprasidone, paliperidone, aripiprazole lauroxil, risperidone, quetiapine, olanzapine, haloperidol, fluphenazine, and valproate. The best response was noted with olanzapine, and the patient had poor adherence to valproate.
• Primary concerns and symptoms: The patient presented with acute‐onset abdominal pain, nausea, and vomiting.

## 3. Clinical Findings

On ED presentation (5/20/2025), the patient was tachycardic (HR 106, BP 117/80), with diffuse mid‐abdominal tenderness and impressive distension without peritonitis. Labs showed hyponatremia (127 mEq/L, chronic baseline) and toxic clozapine level (998 ng/mL). ECG revealed new‐onset atrial fibrillation with RVR. CTA demonstrated pneumatosis intestinalis and portal venous gas. Intraoperative findings showed dilated proximal small bowel with patchy ischemia but no necrosis, perforation, or mechanical obstruction; superior mesenteric artery (SMA) and inferior mesenteric artery (IMA) were palpable with biphasic Doppler signals consistent with hypoperfusion.

## 4. Clinical Timeline

• 3/25/2025: Initiated on clozapine and titrated to 300 mg daily after cross‐tapering from haloperidol decanoate and olanzapine.
• 5/9/2025: Clozapine level 441 ng/mL and norclozapine 288 ng/mL.
• 5/13/2025–5/17/2025: Dose titrated from 300 to 375 mg daily in 25 mg increments every 2 days.
• 5/17/2025: Patient‘s last report of a bowel movement prior to presentation.
• 5/18/2025: The patient developed abdominal pain, nausea, and vomiting.
• 5/20/2025: Presented to ED with abdominal pain, vomiting, and tachycardia; labs showed a toxic clozapine level of 998 ng/mL and norclozapine 552 ng/mL.
• 5/21–22/2025: Underwent emergency laparotomy for suspected bowel obstruction; viable bowel was observed; no resection, reduction, or fixation was performed; atrial fibrillation was observed and resolved postoperatively.
• 5/29/2025: Clozapine/norclozapine levels dropped to the subtherapeutic range after discontinuation.
• 5/30–6/30/2025: Switched back to haloperidol decanoate and olanzapine, with gradual titration back to 15 mg nightly.

## 5. Diagnostic Assessment

• CTA abdomen/pelvis: Showed pneumatosis intestinalis and portal venous gas concerning for bowel ischemia.
• ECG: Atrial fibrillation with RVR.
• Echocardiogram: Normal biventricular size and systolic function; EF 60%–65%; no structural abnormality to suggest a primary cardiac etiology; mild tricuspid regurgitation.
• Surgical findings: Dilated proximal small bowel with patchy ischemia and no evidence of frank necrosis, perforation, or mechanical obstruction; palpable SMA and IMA with biphasic Doppler signals consistent with hypoperfusion etiology.
• Clozapine levels: Abrupt elevation likely secondary to metabolic changes or patient‐specific pharmacokinetic variability [1].
• Basic metabolic panel on admission: Potassium and calcium within normal limits; hyponatremia at 127 mEq/L, consistent with known chronic baseline; no acute electrolyte abnormalities to suggest a primary arrhythmic etiology.
• Lipid panel (6 weeks prior): Elevated triglycerides at 182 mg/dL and total cholesterol 187 mg/dL, consistent with antipsychotic‐associated metabolic dysregulation.

## 6. Therapeutic Intervention

• Psychiatric: Immediate discontinuation of clozapine. Initiated IV haloperidol, later switched to haloperidol decanoate and olanzapine.
• Surgical: Emergency laparotomy; no resection, reduction, or fixation performed; bowel rest was maintained with NPO/NG tube care. A normal diet was resumed once bowel movements returned.
• Cardiology: Managed atrial fibrillation with short‐term amiodarone; no anticoagulation per low CHA2DS2‐VASc score (1 for hypertension).

## 7. Follow‐Up and Outcomes

• Short‐term: No withdrawal symptoms post‐clozapine discontinuation. Atrial fibrillation was resolved postsurgery. Clozapine and norclozapine levels dropped to the subtherapeutic range after discontinuation.
• Long‐term: Recovered gastrointestinally. Psychiatrically, they returned near baseline with residual anxiety and paranoia. Maintained on olanzapine at 15 mg qHS.

## 8. Discussion

This case exemplifies a rare but life‐threatening complication of clozapine therapy, bowel ischemia, with subsequent cardiac dysrhythmia. Elevated clozapine levels here exceeded toxic thresholds, and the patient demonstrated a ~2.3x increase in plasma level within 11 days, highlighting the need for therapeutic drug monitoring, particularly in patients with complex metabolic or pharmacokinetic profiles [1]. The patient in this case report did have risk factors for complication including male gender, being within 4 months of treatment, and a higher daily dose of clozapine [6]. Notably, his symptomatic presentation: sudden onset of abdominal pain, then nausea, and vomiting, prompted ED evaluation within 2 days, underscoring the rapid and severe nature of this adverse event.

Given the controlled nature of the extended acute psychiatric unit where the patient was residing, unsanctioned ingestion of excess clozapine was highly unlikely. Furthermore, several common contributors to clozapine level variability can be reasonably excluded in this context: smoking status was stable throughout the admission, ruling out CYP1A2 induction changes associated with smoking cessation or initiation; no new interacting medications were introduced during this period; adherence variability was minimized given directly observed therapy in the inpatient setting; and there was no documented evidence of hepatic dysfunction [1]. A dose increase from 300 to 375 mg daily was initiated over 4 days following the 5/9 clozapine level and may have partially contributed to the subsequent elevation; however, a 2.3‐fold increase in plasma level within 11 days is disproportionate to the modest dose change alone, and therefore additional pharmacokinetic explanations must be considered. One proposed mechanism, drawn from prior literature rather than confirmed in this case, is the elevation of C‐reactive protein (CRP), a known suppressor of CYP1A2 activity, which in turn reduces clozapine metabolism. CRP levels often increase during systemic inflammation, such as that caused by bowel ischemia and may result in rapid accumulation of clozapine [5]. This hypothesis is supported by prior research indicating elevated CRP as a significant contributor to impaired clozapine clearance and toxicity in medically ill patients [1]. Although CRP was not formally assessed in this case, it is well established that bowel ischemia is associated with systemic inflammatory elevation of CRP, making this mechanism clinically plausible even in the absence of direct measurement [7]. Taken together, CRP‐mediated suppression of CYP1A2 activity in the setting of systemic inflammation, compounded by the recent dose increase, represents the most clinically plausible explanation for the rapid clozapine accumulation observed in this case.

While clozapine‐induced gastrointestinal complications more commonly arise through hypomotility, driven by contributing factors such as sedation, immobility, constipation, and emerging evidence of gut microbiome disruption [5, 6, 8, 9], this case represents a distinct hypoperfusion‐mediated presentation, underscored by the absence of a significant stool burden and the surgical finding of ischemia without mechanical obstruction. This is unlike prior reported cases which describe hypomotility‐driven adynamic obstruction or perforation secondary to elevated intraluminal pressure [10, 11]. Bowel ischemia due to hypoperfusion or microvascular effects may result from anticholinergic and antiserotonergic‐induced motility disturbances and vascular changes, a phenomenon rarely reported but increasingly acknowledged [5, 10, 12–15]. Clozapine, along with other antipsychotics, has been associated in the literature with metabolic disturbances including hyperlipidemia, which may contribute to endothelial dysfunction and impaired microvascular perfusion [2, 3, 16]. Consistent with this, a lipid panel obtained approximately 6 weeks prior to the ischemic event demonstrated elevated triglycerides at 182 mg/dL, suggesting that antipsychotic‐associated metabolic dysregulation may have contributed to increased susceptibility to microvascular ischemia in this patient. Furthermore, complete remission of bowel ischemia following clozapine discontinuation, as reported in the literature, aligns with our patient’s trajectory [13].

To formally assess causality, the Naranjo Adverse Drug Reaction Probability Scale was applied [17, 18]. The patient received a score of +8, which was consistent with a probable adverse drug reaction. Points were assigned for prior literature documenting this reaction (+1), confirmed temporal association (+2), clinical improvement following clozapine discontinuation (+1), toxic drug levels confirmed on laboratory testing (+1), objective confirmation via imaging and surgical findings (+1), and exclusion of alternative etiologies (+2). Substance use‐related vasospasm was excluded given the controlled inpatient setting, and hemodynamic instability was excluded given the documented normal blood pressure throughout the admission. While underlying vascular disease cannot be entirely excluded, the intraoperative finding of palpable SMA and IMA with biphasic Doppler signals argues against significant large vessel occlusion, and the diffuse pattern of ischemia is inconsistent with a primary vascular etiology.

The resolution of AF after ischemia supports a reactive cardiac response to systemic inflammation or hypoxia, not a primary embolic etiology. Cardiology and radiology teams agreed that the ischemia was diffuse rather than localized to a specific vascular territory, and no other intra‐abdominal embolic sites were identified on imaging. Echocardiography demonstrated normal biventricular size and systolic function with an ejection fraction of 60%–65% and no structural abnormality to suggest a primary cardiac etiology, further supporting a reactive rather than structural or embolic mechanism for atrial fibrillation. Electrolytes on admission did not reveal acute abnormalities to suggest a primary electrolyte‐driven arrhythmia, with potassium and calcium within normal limits and hyponatremia consistent with the patient’s known chronic baseline. The combination of a structurally normal heart, diffuse rather than focal ischemia on imaging, and spontaneous resolution of AF following surgical management is consistent with a secondary reactive dysrhythmia in the setting of systemic inflammation and physiologic stress response to bowel ischemia [19]. Most plausibly, bowel ischemia precipitated reactive atrial fibrillation rather than being a consequence of a primary cardiac event.

Given that clozapine‐induced gastrointestinal complications may be clinically silent in the early stages and can progress rapidly to life‐threatening presentations, practical vigilance is warranted. Routine therapeutic drug monitoring of clozapine plasma levels is essential, with heightened frequency during intercurrent illness, systemic inflammation, or any suspected pharmacokinetic change. New gastrointestinal symptoms including abdominal pain, nausea, or vomiting in clozapine‐treated patients warrant prompt evaluation, with a low threshold for advanced imaging given the potential for rapid deterioration. Clinicians should also be aware that toxic clozapine levels may develop secondary to reduced CYP1A2 activity during inflammatory states and may exacerbate the effects of even modest dose increases, as observed in this case. Early involvement of surgical and medical teams when ischemia is suspected is critical as delayed intervention significantly worsens outcomes [5, 6, 12, 13]. When transitioning away from clozapine following such complications, antipsychotic selection warrants careful consideration as bowel ischemia has also been reported with olanzapine [20].

## 9. Conclusion

Clinicians prescribing clozapine should maintain high vigilance for rare gastrointestinal and cardiovascular adverse effects. Early signs such as GI discomfort or unexplained tachycardia warrant prompt evaluation. Importantly, gastrointestinal complications may arise through hypoperfusion mechanisms in the absence of hypomotility or mechanical obstruction, and clinicians should not anchor solely on hypomotility‐driven etiologies when evaluating clozapine‐treated patients with abdominal symptoms. This case reinforces the need for routine clozapine level monitoring and individualized risk–benefit evaluation in complex psychiatric patients.

## Funding

No funding was received for this study.

## Ethics Statement

This case report was reviewed and determined to be exempt from IRB review per Temple University LKSOM policy as it represents a clinical observation from routine patient care rather than prospective research.

## Consent

Written informed consent was obtained from the patient for the publication of this case.

## Conflicts of Interest

The authors declare no conflicts of interest.

## Data Availability

Data sharing is not applicable to this article as no datasets were generated or analyzed during the current study.
